# Income Inequality in Dental Visits Among Chinese Children: Analysis of Longitudinal Data

**DOI:** 10.7759/cureus.72839

**Published:** 2024-11-01

**Authors:** YuXuan He, Anqi Shen, Wael Sabbah

**Affiliations:** 1 Dental Public Health, Faculty of Dentistry, Oral and Craniofacial Sciences, King's College London, London, GBR; 2 Department of Preventive Dentistry, Beijing Stomatology Hospital, Capital Medical University, Beijing, CHN

**Keywords:** children’s caries, dental visits, income inequality, longitudinal study, oral health

## Abstract

Aim: This study aimed to explore whether income inequality exists in dental visits among children when children/parents were informed they had caries.

Methods: This study used data from a prior longitudinal study carried out in Liaoning Province, China, involving 772 children under the age of five from local kindergartens. Children were clinically assessed for dental caries at baseline, data on dental visits were collected a year later. Logistic regression model was used to analyze the association between dental caries and household income at baseline and dental visits at follow-up, a year later.

Results: In the logistic regression analysis accounting for age and sex, the results showed that household income and dental caries had significant association with dental visits among Chinese children. Lower household income was negatively associated with dental visits, with those at the lowest income level having lower odds ratio (OR) of 0.27 (95% confidence interval (CI): 0.16, 0.47), and the second lowest income level, with OR of 0.39 (95% CI: 0.22, 0.71) compared to highest-income group. There was a statistically significant association between dental caries at baseline and dental visits in the following year (OR: 1.08, 95% CI: 1.02, 1.15).

Conclusion: There were income inequalities in dental visits among children in China, even when they were diagnosed with dental caries. Socioeconomic inequality appears to be a major barrier to dental visits among Chinese children with dental needs.

## Introduction

Dental caries continuously increases as a global public health problem, leading to significant economic burdens. In 2022, the WHO showed that there were two billion people and 514 million children with dental caries [1]. Dental caries can cause negative physical and psychological consequences, especially in children.

In China, according to the Fourth National Oral Health Survey, about 63.11% of children suffer from early childhood caries (ECC) [2]. Zou et al. [3] reported that 97% of caries had not been treated appropriately among children. These figures are much worse than in some developed countries; for example, in the USA, only 23% of preschool children had dental caries in their primary teeth [4]. Children’s caries can negatively impact children’s quality of life, impairing pronunciation, mastication, aesthetics, and causing economic burdens for many families at the same time [2]. Thus, dental caries among Chinese children is a notable health issue that requires continuous focus and action from the Chinese government.

Qu et al. [5] reported that children who maintained routine dental examinations had a lower incidence of caries than those who did not have regular dental visits. Therefore, access to dental services helps in prevention, early diagnosis, and timely intervention of early childhood caries [6]. In the USA, 41.9% of children visit the dentist annually. Meanwhile, in Belgium, 79% of preschool children have used dental services [7]. However, the use of dental services in China is not prevalent as compared to the developed countries. According to the Fourth National Oral Health Epidemiological Survey, only 14.6% of children (three to five years old) sought dental care [8], which may be attributed to the Chinese inadequate health insurance coverage and payment system, especially for dental expenditure. Although the Chinese government has implemented four main health insurance schemes, dental services are still not freely available in China. Different socioeconomic indicators and affordability of care influence health insurance options and lead to inequality in access [9]. However, despite the support of health insurance, there are still almost 85% of expenditures of dental services paid by individuals [10,11]. Some indirect costs, such as time off work and cost of transportation, can also negatively affect the willingness to utilize dental services. These factors affect the ability of those with dental problem to seek dental care and contribute to socioeconomic inequalities in caries and in dental visits.

This study aimed to examine whether there is socioeconomic inequality in dental visits among Chinese children, regardless of the presence of caries. The objective was to assess the association between household income and dental visits among Chinese children.

## Materials and methods

Ethical approval

The original study was ethically approved by the Ethics Committee of King’s College London (KCL; ref: HR-15/16-2901), and Shenyang Dental Hospital gave oral consent (Ministry of Health of People’s Republic of China) [12]. All the participants’ parents agreed to take part in the study and signed informed consent. 

Study population

This is a secondary analysis of data from a longitudinal population-based study conducted in Shenyang, Liaoyang, and Fushun from Liaoning Province, in China, between 2016 and 2017 [13]. Inclusion criteria were children enrolled in kindergartens in Shenyang, Liaoyang, and Fushun, Liaoning province, age two to five years. Exclusion criteria were children whose parents did not consent to participation in the study. At baseline, 17 kindergartens were approached, and only 15 agreed to participate. At baseline, a total of 15 kindergartens were included in the study, and 1111 children (≤5 years old) participated in the survey. During the one year follow-up, 339 children withdrew from the survey, mainly due to changing kindergarten or moving out of the city. Therefore, the final response rate was nearly 70%.

Data collection

Shen et al. [13] included all participants aged zero to five years old (N = 772) who completed the whole survey at baseline and follow-up. They collected data on sex, age in months, the number of decayed and filled teeth (dft), household income, mother’s education, dietary habits, and dental visits. The dft was assessed by one dentist, using the WHO standard criteria. Intra-examiner reliability was assessed by re-examining 80 children, agreement beyond chance (Kappa) was 0.72. Household income was divided into quartiles: lowest (0-3999 RMB), second lowest (4000-5999 RMB), second highest (6000-9999 RMB), and highest (10,000 RMB or above) [13], according to the Fourth Chinese Oral Health Survey. 

Dental caries was categorized into two groups: no caries or filling (dft = 0) and any caries or filling (dft > 0) [2]. Dental visits were also categorized as a binary variable (0: no dental visits within the follow-up time, 1: had at least one dental visit). Household income was used as an indicator of socioeconomic condition. 

Data analysis

We used Stata Statistical Software (StataCorp., College Station, TX) for data analysis. Significance level was set on <0.05. Age in months was used as a continuous variable, and all other variables were categorical. First, we examined the characteristics of the population in terms of age, sex, dft, and socioeconomic factors (household income) at baseline. The distribution of dental visits at follow-up and the prevalence of caries at baseline were examined within each variable included in the study. Chi-square and T-test were used to asses the relationships between age, sex, and income with each of dental visits and dental caries, respectively. Furthermore, the association between dental visits at follow-up and household income was assessed among children with caries at baseline.

Given that the outcome was a binary variable and to account for confounding factors, logistic regression was used to assess the relationship between dental visits during follow-up time and household income adjusting for age, sex, and dental caries. An additional model only included children with caries at baseline and adjusted for the same variables as the first model. All analysis was done using STATA software.

## Results

During follow-up, there were 339 participants with missing data as they left the study during follow-up period. Finally, 772 preschool children from Liaoning Province were enrolled in the analysis.

Table 1 shows that the mean age of the participants at baseline was 50.8 months. There were 51.4% boys and 48.6% girls, respectively. At baseline, 58.0% of children had decayed teeth. However, only 20.2% of reported to visiting a dentist in the following year. Dental caries was the lowest among children from the highest-income group (43.4%), but dental visits was the highest among this group (31.5%) compared to lower-income groups (Table 1).

**Table 1:** Descriptive characteristics of Chinese preschool children, adjusted by dental visits and caries at baseline (N = 772) * *p*-value from the chi-square/T-test; *p < 0.05, **p < 0.01, ***p < 0.001; N/A: not applicable

Variables				Percentage/mean (95% CI)	Percentage/mean who visited a dentist	Percentage/mean with caries at baseline
Age (months)		Visited a dentist/with caries at baseline		50.8 (50.1, 51.6)	52.6^*^ (51.2, 54.1)	53.3^***^ (52.4, 54.2)
		No dental visits/no caries at baseline			50.4 (49.5, 51.2)	47.4 (46.3, 48.5)
Sex	Male			51.4% (47.9, 55.0)	18.1%	55.7%
	Female			48.6% (45.1, 52.1)	22.4%	60.5%
Income	Lowest			44.0% (40.6, 47.6)	13.8%^***^	61.5%^**^
	Second Lowest			21.0% (18.3, 24.1)	18.5%	65.4%
	Second Highest			21.0% (18.3, 24.0)	27.8%	53.1%
	Highest			14.0% (11.7, 16.6)	31.5%	43.5%
Dental visits within the last year			Visited a dentist	20.2% (17.5, 23.2)	N/A	76.9%^***^
			No dental visits		N/A	53.3%
Prevalence of dental caries at baseline				58.0% (54.5, 61.5)	N/A	N/A
Mean number of caries at baseline			Mean with dental visits	3.0 (2.8, 3.3)	4.5^***^ (3.9, 5.2)	N/A
			Mean without dental visits		2.7 (2.4, 2.9)	N/A

**Figure 1:**
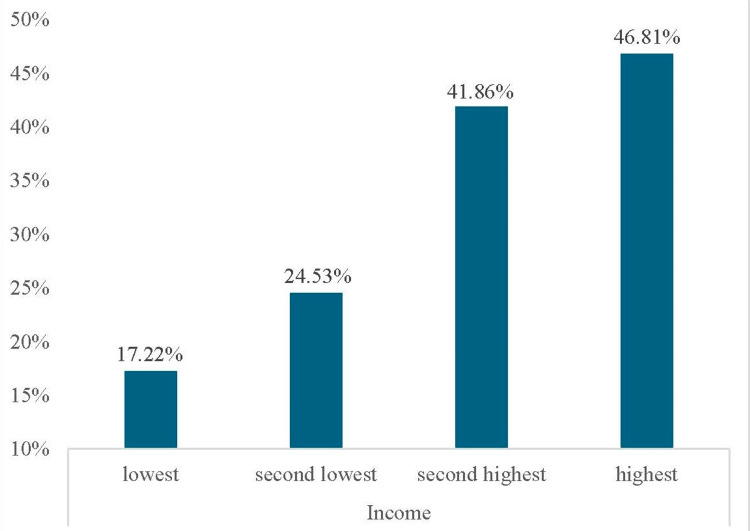
Different proportions of dental visits among children with caries impacted by household income

Figure 1 shows the prevalence of dental visits by household income among children who had caries at baseline. The figure demonstrates a clear income gradient in dental visits among children with caries.

Table 2 shows the association between household income and dental visits adjusting for age, gender, and dental number of teeth with filling or caries (dft). Lower income were negatively and significantly associated with dental visits with odds ratios (ORs) of 0.27 (95% CI: 0.16, 0.47) and 0.39 (95% CI: 0.22, 0.71) among children in the lowest and second-lowest-income groups, respectively, compared to those in highest-income group (Table 2, Model 1).

**Table 2:** Logistic regression of factors associated with dental visits within the last year among preschool children in China *p < 0.05, **p < 0.01, ***p < 0.001 Model 1 adjusted for age, sex, income, and dental caries for the whole sample (N = 772). Model 2 includes children with caries at baseline (N = 448).

		Model 1	Model 2
Variables		Odds ratio (95%CI)	Odds ratio (95%CI)
Age (months)		1.01 (0.99, 1.03)	1.00 (0.97, 1.02)
Sex (ref: male)	Female	1.19 (0.82, 1.72)	1.23 (0.79, 1.91)
Income level (ref: highest quartile)	Second highest	0.78 (0.45, 1.36)	0.81 (0.39, 1.67)
	Second lowest	0.39^**^ (0.22, 0,71)	0.36^**^ (0.17, 0.74)
	Lowest	0.27^***^ (0.16, 0.47)	0.23^***^ (0.11, 0.45)
Dental caries		1.14^***^ (1.08, 1.19)	1.08^*^ (1.02, 1.15)

When we limited the analysis to those who had caries at baseline, the ORs were 0.23 (95% CI: 0.11, 0.45) and 0.36 (95% CI: 0.17, 0.74) for the lowest and second-lowest groups, respectively, compared to the highest-income group (Table 2, Model 2).

## Discussion

This study assessed the relationship between household income and dental visits among Chinese children who were diagnosed with caries a year earlier. The results demonstrated that lower income levels were negatively and significantly associated with dental visits. Although a large number of children had tooth decay, especially in the lowest-income families, the lowest percentage of children from that income group reported dental visits. 

Childhood caries is socially patterned with those at the lower end of social hierarchy experiencing higher levels of the disease, regardless of the indicator of socioeconomic factors [14]. Furthermore, behavioral and dietary risk factors for children’s caries are also socially patterned, which exacerbate inequalities in children caries. These inequalities do not only exist within countries but also between countries. Vukovic et al. [15] demonstrated that nearly 90% of children living in higher-income countries had better oral health than those living in poorer countries. 

On the other hand, timely dental visits are essential for prevention, early detection, and treatment of children’s caries. Both the American Academy of Pediatric Dentistry (AAPD) in America and the American Dental Association (ADA) have emphasized the significance of routine dental visits [16,17]. In North Carolina, Beil et al. [18] concluded that early dental visits can help keep this oral disease at bay in children at high risk for caries. However, the study findings indicated that children from affluent households had a lower occurrence of dental caries and higher level of dental visit, a finding similar to that reported in this study among Chinese children. Although earlier research suggested that the presence of caries would encourage children to visit dentists despite the financial barriers [18], this does not seem to be the case among Chinese children. There are several barriers to dental visits among children in China. First is the cost of the service, which is relevant to income and ability to pay [19]. Health insurance in China currently covers only the most basic dental treatment. About 85% of the cost needs to be provided by the patient [10,11,20]. Undoubtedly, this contributed to low dental visits, with only 13.1% of children in 2019 [21]; many families in China cannot afford regular visits to the dentist without financial support. The financial barriers for dental visits, particularly among children from low socioeconomic families, contribute to the growing problem of children’s caries in China. This is particularly important as various studies reported how poorer oral hygiene and oral habits [22,23] contribute to increases in caries levels among children. Furthermore, with the dietary transition in China [24], there is also higher consumption of sugars, which also contribute to the problem of dental caries [25].

While the recent policy in China, "Healthy China 2030 Blueprint," aims to improve access to dental services [26] and provide preventive dental services [11], other policies relevant to sugar intake could also be implemented. For example, the UK and USA governments have introduced sugar taxation to reduce use of sugar sweetened beverages [27,28]. These policies could also be beneficial if implemented in China.

The strengths of this study is in using longitudinal data and assessing inequalities in dental visits a year after the children were told they had untreated caries. Furthermore, the study demonstrated income inequality among those who had caries at baseline. The study has few limitations worth mentioning. First, children who attend kindergartens, selected in this study, are usually from more affluent families. This indicates that the inequalities in dental visits could be underestimated [12]. Second, there are other factors that could contribute to dental visits not included here, such as taking time off work, transportation (indirect cost of dental visits), and dental fear and anxiety.

## Conclusions

In this study, longitudinal data from Liaoning Province, in China, were used to assess inequality in dental visits among children between two and five years. The survey used data on dental caries at baseline and dental visits during the following year. Household income at baseline was used to assess inequalities. While the occurrence of caries increases the need for dental care, many Chinese children could not visit a dentist even after being diagnosed with caries a year earlier. The analysis highlights income inequalities in dental visits among Chinese children. Even when the analysis was limited to those diagnosed with caries, inequalities in dental visits persisted. The findings highlight the importance of introducing universal coverage for oral healthcare, particularly for children. The findings also emphasise the need for effective health promotion policies and preventive interventions to reduce the burden of dental caries.
